# Editorial: Leveraging artificial intelligence and open science for toxicological risk assessment

**DOI:** 10.3389/ftox.2025.1568453

**Published:** 2025-02-12

**Authors:** Marc Teunis, Thomas Luechtefeld, Thomas Hartung

**Affiliations:** ^1^ Department of Life Sciences and Chemistry, HU—University of Applied Sciences Utrecht, Utrecht, Netherlands; ^2^ Insilica, LLC, Bethesda, MD, United States; ^3^ Johns Hopkins University, Bloomberg School of Public Health and Whiting School of Engineering, Baltimore, MD, United States; ^4^ Department of Biology, University of Konstanz, Konstanz, Germany

**Keywords:** artificial intelligence, machine learning, toxicology, risk assessment, open science, computational toxicology, predictive modeling

The paradigm shift brought about by artificial intelligence (AI) across scientific disciplines has been nothing short of revolutionary. From unraveling the mysteries of protein folding to enabling autonomous systems, AI has demonstrated its potential to tackle previously intractable problems (Jumper et al., 2021; Abramoff et al., 2023). In toxicology, this transformation arrives at a crucial moment, as we face mounting challenges in chemical safety assessment and an urgent need to reduce reliance on animal testing (Hartung, 2023a; Hartung, 2023b; Kleinstreuer and Hartung, 2024).

This Research Topic emerged from a recognition that while computational toxicology has made significant strides using classical approaches such as physiologically-based pharmacokinetic (PBPK) modeling and quantitative structure-activity relationships (QSAR), the full potential of modern AI techniques remains largely untapped in toxicological risk assessment. The recent advances in machine learning, particularly deep learning, natural language processing, and semantic interoperability, offer unprecedented opportunities to integrate diverse data sources and create more predictive models for human health outcomes.

The five contributions in this Research Topic showcase innovative approaches that bridge traditional toxicological methods with cutting-edge AI applications. Collectively, these works demonstrate the potential for AI to enhance our understanding of chemical hazards while advancing the development of more efficient, ethical, and human-relevant risk assessment strategies.

Vikram et al. “*Predicting Carcinogenic Risk from Chemical-Induced Genomic Instability*” This review highlights the potential of AI/ML and OMICS technologies to transform traditional toxicological assessments and predict genotoxicity and mutagenicity with higher accuracy. It discusses how AI/ML can establish biomarkers and signatures for early cancer detection, risk assessment, and monitoring of health impacts from chemical exposure. Additionally, it emphasizes how AI may accelerate the screening of chemicals for toxicological evaluation, optimizing resource use, and reducing reliance on animal testing.

Snyder et al. “*sendigR: an R package to leverage the value of CDSIC SEND datasets for cross-study analysis*” This paper introduces the “sendigR” R package, developed to facilitate cross-study analysis of nonclinical toxicology study data. It provides functions to build and query a relational SEND database and offers a user-friendly web interface for historical control data analyses. The package supports advanced analyses via custom SQL queries and can serve as a foundation for developing user-friendly web-based applications to assess the safety of investigational therapeutics.

Odje et al. “*Unleashing the Potential of Cell Painting Assays for Compound Activity and Hazard Prediction*” This review explores the use of Cell Painting (CP) assays in drug discovery and toxicology. It discusses integrating CP-based phenotypic data with compound structures into machine learning models to predict activities for various disease endpoints and identify underlying modes of action, all while reducing animal testing.

Corradi et al. “*The application of Natural Language Processing for the extraction of mechanistic information in toxicology*” This paper demonstrates the use of Natural Language Processing (NLP) to extract information from scientific texts, speeding up reviews and discoveries. It applies NLP to select chemicals for testing toxicological endpoints and construct adverse outcome pathways. The authors present a proof-of-concept for NLP in toxicology and offer an open-source model for recognizing toxicological entities and their relationships.

Trovato et al. “*Cross clinical-experimental-computational qualification of in silico drug trials on human cardiac Purkinje cells for pro-arrhythmia risk prediction*” This study quantifies drug-induced electrophysiological effects on *in silico* human cardiac Purkinje cells, comparing them with *in vitro* rabbit data to assess their accuracy in predicting clinical pro-arrhythmia risk. It demonstrates the higher accuracy of *in silico* methods compared to *in vitro* animal models for proarrhythmic risk prediction and highlights the consistency with *in vitro* experiments used in safety pharmacology.

A common thread throughout these contributions is the emphasis on open science principles and reproducibility. The authors have shared their code, data, and methodologies through public repositories, enabling others to build upon their work. This commitment to transparency and collaboration exemplifies the transformation that AI is bringing not only to scientific problem-solving but also to the way we conduct and share research.

Looking ahead, several challenges and opportunities emerge from these works. First, while AI shows promise in predicting toxicological endpoints, integrating these predictions into regulatory frameworks remains a significant hurdle (Hartung and Kleinstreuer, 2025). Second, the quality and accessibility of training data continue to be limiting factors in developing robust AI models. Finally, there is a pressing need for standardized approaches to validate AI-driven predictions in toxicology.

Nevertheless, the works presented in this Research Topic demonstrate that we are moving closer to establishing a true probabilistic risk assessment framework (Maertens et al., 2022; Maertens et al., 2024) that incorporates the full potential of AI. Such a framework could not only reduce animal testing but also provide more accurate predictions of human health effects. The integration of multiple data streams - from chemical structures to historical animal data, *in vitro* assays, and omics measurements - through AI approaches represents a promising path forward.

We hope this Research Topic will serve as both an inspiration and a practical resource for researchers working at the intersection of AI and toxicology. As we continue to advance these methods, we move closer to more efficient, ethical, and accurate approaches to chemical safety assessment.

## Abbreviations

AI, Artificial Intelligence; CDISC, Clinical Data Interchange Standards Consortium; CP, Cell Painting; ML, Machine Learning; NLP, Natural Language Processing; OMICS, The collective methods for studying the whole of biological molecules on organismal or population level; PBPK, Physiology-Based Pharmaco Kinetic(s); QSAR, Quantitative Structure-Activity Relationships; SEND, Standard for the Exchange of Nonclinical Data; SQL, Structured Query Language.
